# Self-Regulatory Processes, Motivation to Conserve Resources and Activity Levels in People With Chronic Pain: A Series of Digital N-of-1 Observational Studies

**DOI:** 10.3389/fpsyg.2020.516485

**Published:** 2020-09-04

**Authors:** Gail McMillan, Diane Dixon

**Affiliations:** ^1^Department of Psychology, Carleton University, Ottawa, ON, Canada; ^2^Institute of Applied Health Sciences, University of Aberdeen, Aberdeen, United Kingdom

**Keywords:** chronic pain, N-of-1, digital health, self-regulation, self-regulatory fatigue, motivation

## Abstract

**Objectives:**

Motivational and self-regulatory processes during goal pursuit may account for activity patterns in people with chronic pain. This article describes a series of N-of-1 observational studies designed to investigate the influence of goal-related factors on fluctuations in motivation to conserve resources and objectively measured activity levels.

**Methods:**

Four participants with chronic pain who attended a formal pain management program (PMP; 41–59 years old; three female) were recruited and completed digital daily diaries for 11–12 weeks. The daily dairies, delivered via text message, measured self-regulatory fatigue, goal self-efficacy, goal striving, perceived demands, pain, and motivation to conserve resources. Continuously worn accelerometers measured physical activity and sedentary time. Analyses were conducted individually for each participant. The effects of self-regulatory fatigue, goal self-efficacy, goal striving, perceived demands, and pain on motivation to conserve resources, physical activity and sedentary time were assessed with dynamic regression modeling.

**Results:**

Different patterns of associations between the predictors and outcomes were observed across participants. Most associations occurred concurrently (e.g., on the same day). Perceived demand was the only variable to predict motivation to conserve resources, physical activity, and sedentary time. Motivation to conserve resources and sedentary time were most frequently predicted by goal striving and perceived demand. Self-regulatory fatigue and pain intensity both predicted motivation to conserve resources in two participants and sedentary time in one participant. Motivation to conserve resources predicted sedentary time in two participants.

**Conclusion:**

This study was the first to examine the impact of fluctuations in self-regulatory processes on motivation to conserve resources and objective activity levels within individuals with chronic pain. The results generally supported recent affective-motivational views of goal pursuit in chronic pain. This study demonstrated that N-of-1 observational studies can be conducted with patients during a PMP using digital technologies. The use of these approaches may facilitate the application of personalized medicine.

## Introduction

Both underactivity and overactivity patterns have previously been deemed maladaptive and implicated in the maintenance and exacerbation of chronic pain (Philips, 1987; Vlaeyen and Linton, 2000, 2012; Hasenbring and Verbunt, 2010; Hasenbring et al., 2012). Underactivity, also known as pain avoidance behavior, is defined as a decrease in general daily activity and physical activity (Vlaeyen and Crombez, 1999). Overactivity, also known as persistence or endurance behavior, can increase pain and lead to long, inactive recovery periods or a “yo-yo” pattern of activity (Fordyce, 1976; Nielson et al., 2013). More recently, the utility of describing underactivity or overactivity as maladaptive has been challenged. It seems that only a small subset of people with chronic pain reduce activity levels (Bousema et al., 2007; Pincus et al., 2010; van Weering et al., 2011). Meanwhile, there is still ambiguity as to when endurance behavior can be detrimental or advantageous (Kindermans et al., 2011; Andrews et al., 2012; Hasenbring et al., 2012).

The reasons *why* individuals with chronic pain engage in different activity patterns may be more important than the patterns themselves. The interruptive nature of pain is often considered a barrier to engaging in valued activities and goals in people with chronic pain (Affleck et al., 1998; Eccleston and Crombez, 1999; Karoly and Ruehlman, 2007; Bushnell et al., 2013). However, the psychosocial, motivational and affective context of pursuing valued goals and activities must be examined (Crombez et al., 2012; Murphy, 2015; Van Damme and Kindermans, 2015). That is, the adoption of different physical activity patterns depends on the individual context of goal pursuit.

The Goal Centered, Self-regulatory, Automated, Social Systems Psychology (GRASSP) model (Karoly, 2010, 2018) is an integrative motivational model that assumes that goal pursuit in people chronic pain is accounted for by day-to-day goal-guided self-regulatory processes, neurobiological factors, and the individual psychosocial, motivational and affective context. The experience of chronic pain determines motivation, or *goal directedness*, by impacting goal-related thoughts, feelings and striving, and capacity to engage in self-regulatory efforts and strategies (Karoly, 2018). According to the GRASSP model, motivation during goal pursuit episodes is impacted by altering the value of activities and the cost-benefit analysis of engaging in activities (Karoly, 2018). Given that perceived demands of activities are considered in a cost-benefit analysis of goal pursuit, perceived demands may be directly related to motivation to conserve resources and activity levels. In addition to factors which undermine motivational and self-regulatory processes, GRASSP considers motivational buffers which facilitate goal-striving (Karoly, 2018). Most notably, self-efficacy, confidence in one’s ability to complete a task (Bandura, 1977), is implicated in the allocation and conservation of resources during goal pursuit in people with chronic pain, facilitating or inhibiting goal striving (Karoly, 2018).

The capacity to engage in self-regulatory effort and strategies in people with chronic pain is affected by self-regulatory fatigue (Solberg Nes et al., 2010; Karoly, 2018). Self-regulatory fatigue is a decrease in general self-regulatory capacity, meaning self-regulation in cognitive, emotional and behavioral domains are more taxing and less effective (Solberg Nes et al., 2010, 2011). Experimental methods have demonstrated that people with chronic pain have lower self-regulatory capacity than healthy controls, resulting in poorer self-regulatory performance (Solberg Nes et al., 2010, 2011). People with chronic pain also have lower heart rate variability, a physiological indicator of lower self-regulatory capacity, compared to healthy controls (Koenig et al., 2016; Rost et al., 2017). Self-regulatory fatigue impacts motivation in people with chronic pain by increasing motivation to conserve resources (Hobfoll, 1989; Muraven et al., 2006; Eisenlohr-Moul et al., 2013). Pain intensity has a dose dependent effect on self-regulatory performance, where higher pain was associated with poorer performance (Solberg Nes et al., 2010).

An examination of the role of fluctuations in self-regulatory processes including self-regulatory fatigue, pain, self-efficacy perceived demands, and goal striving on motivation to conserve resources and activity patterns in people with chronic pain will further our understanding of mechanisms of goal pursuit. Investigating the dynamic pursuit of valued personal goals and their determinants has been identified as an important line of research for understanding the effects of pain in the broader context of living a meaningful life (Winger et al., 2019). Yet, the majority of past research with clinical samples has relied on pre-post intervention assessments with retrospective self-report, which are subject to recall and error biases (Stone et al., 2003, 2004, 2005; Stone and Broderick, 2007; Broderick et al., 2008). These approaches have not captured the dynamic nature of motivational processes of pursuing goals in daily life while living with chronic pain (Karoly, 2018; Mun et al., 2019). Self-regulation is a dynamic process, which requires dynamic measurement (Neal et al., 2017). Therefore, using methods that observe dynamic fluctuations in pain, motivation, and self-regulatory processes over time within-person are needed.

N-of-1 designs, which involve intensive longitudinal repeated measurement within an individual, are one such method of assessing within-person variability (Johnston and Johnston, 2013). These designs allow conclusions to be drawn about intraindividual variation over time which will advance the science of pain dynamics (Karoly, 2018; Mun et al., 2019). It has been recommended that N-of-1 methods are used to test theory and interventions (Craig et al., 2008; McDonald et al., 2017a; Kwasnicka and Naughton, 2020). For example, N-of-1 methods have been used to assess whether social cognitive constructs predict physical activity within individuals (Hobbs et al., 2013; O’Brien et al., 2016; McDonald et al., 2017b; Smith et al., 2019; Kwasnicka and Naughton, 2020).

The capability to evaluate the dynamic processes of pain and motivation has been facilitated tremendously by developments in digital health methodologies. The ability of text messaging, mHealth applications (apps) and wearable devices to provide precise, real-time observations of physical (e.g., pain), psychological (e.g., self-efficacy), physiological (e.g., heart rate), and exogenous (e.g., day of the week and weather) variables provides real opportunity to reduce recall biases and burden for participants (Winger et al., 2019). Thus, digital health technologies facilitate the collection of more ecologically valid data. Moreover, the use of multiple digital health technologies simultaneously (e.g., wearable accelerometers, heart rate devices, and recording of cognitions through a smartphone) allows for a holistic bio-psychosocial approach to be taken to data collection and the subsequent design of interventions (Marceglia and Conti, 2017; Mun et al., 2019; Winger et al., 2019).

Understanding dynamic motivational processes via digital health technologies can have a direct impact on treatment for people with chronic pain. An advantage of digital health technologies is that data-driven, individual treatment plans can be easily accessed by the majority of the population at low cost (Marceglia and Conti, 2017). mHealth apps accessible to patients via their smartphones provide the opportunity for patients to self-monitor and gain insights which facilitate behavior change and self-management (Aaron et al., 2005), which is the ultimate goal of treatment for chronic pain. Real-time recording through digital health technologies also provides both patients and healthcare providers with detailed reports of progress and obstacles (Winger et al., 2019). Furthermore, when designing interventions to increase physical activity, taking a personalized approach may yield better results (Noar et al., 2007; Hobbs et al., 2013). Particularly, personalized, data-driven pacing plans in people with chronic pain may be of particular benefit (Murphy et al., 2010; Murphy, 2015).

Therefore, using a combination of a digital daily diary method and wearable accelerometer devices, the aim of the present study was to examine the effect of variation in self-regulatory process during goal pursuit. The effects of self-regulatory fatigue, goal self-efficacy, pain, goal striving and perceived demands on motivation to conserve resources, physical activity and sedentary time during daily living were examined in individuals with chronic pain. Based on between-person group-level studies, it would be expected that self-regulatory fatigue, pain and perceived demands predict motivation to conserve resources and sedentary time, while negatively predicting physical activity. It is hypothesized that goal self-efficacy and goal striving would be negatively related to motivation to conserve resources and sedentary time while being positively related to physical activity.

## Methods

### Design

A series of N-of-1 observational studies were conducted for approximately 84 days (12 weeks) over the duration of a Pain Management Program (PMP). A digital daily diary method was used to measure study variables by self-report twice daily, once in the morning (between 7 am and 10 am) and again 12 h later. Therefore, there were around 168 observations in total for each participant on each variable (84 in the morning and 84 in the evening).

### Participants

Participants who were due to attend a National Health Service (NHS) based PMP in Scotland were recruited by clinician referral. Inclusion criteria for this study were that patients were between the age of 18 and 65 years old, experienced chronic pain (defined as persistent pain lasting longer that 3 months), fluent in the English language, not currently experiencing acute injury and that they were due to begin the PMP within 3 months. Patients who were interested in participation were provided a letter of invitation and information about the study. Patients who expressed an interest were given a 1-week consideration period, and were then contacted and invited to participate in the study. Seven participants (six female and one male) were invited to take part. Of those seven invitees, one decided not to take part prior to the baseline meeting and one participant had to withdraw as they could not commence the PMP until after the data collection period would end. Another participant began the study but withdrew less than half-way through the PMP and a technical issue compromised their evening data collection meaning the available data could not be examined. Therefore, four participants completed the study. The study was granted ethical approval by the NHS South West-Central Bristol Research Ethics Committee (reference number: 18/SW/0076).

### Measures

#### Baseline

##### Demographics

Each participant provided their age and gender. Participants were asked to describe any physical or mental health conditions they were experiencing.

##### Pain

Participants provided the duration of their pain (years). Current and average pain (pain over the past 6 months) intensity was rated on an 11-point Likert scale from 0 (no pain) to 10 (pain as bad as can be). Measuring current pain intensity by numerical rating scale is a valid, reliable and sensitive method of assessing present pain level (Williamson and Hoggart, 2005; Ferreira-Valente et al., 2011).

##### Physical functioning

Physical functioning was assessed by self-report using the PROMIS Physical Function Short Form 8a (PROMIS PF-8a). The PROMIS PF-8a (Cella et al., 2010) is an eight item measure developed from the PROMIS items bank of 124 physical functioning items which measure mobility, dexterity, movement of neck and back, and instrumental activities. The PROMIS PF-8a assesses current ability to perform basic activities of daily living. Four items on the measure (e.g., “Are you able to go up and down stairs at a normal pace”; “Are you able to run errands and shop?”) are rate on a 5-point Likert scale anchored by 5 (“Without any difficulty”) to 1 (“Unable to do”). Four items (e.g., “Does your health now limit you from doing 2 h of physical labor?”; “Does your health now limit you in lifting and carrying groceries?”) are measured on a 5-point Likert scale anchored by 5 (“Not at all”) to 1 (“Cannot do”). All items are summed and the scale provides a score range of 8–40 where higher scores indicate better physical functioning.

##### Self-regulatory fatigue

The Self-regulatory Fatigue Scale (Solberg Nes et al., 2013) measures self-regulation fatigue, or a reduced capacity to self-regulate, in chronic multisymptom illness (e.g., “It is easy for me to set goals”). Each item is scored on a 5-point Likert scale from strongly disagree to strongly agree. The scale measures cognitive (6 items), emotional (7 items) and behavioral (5 items) components of self-regulatory fatigue to produce an 18-item scale with a range of 18–90 where higher scores indicate higher self-regulatory fatigue.

##### Pain self-efficacy

The Pain Self-efficacy Questionnaire (Nicholas, 1989) measures confidence in ability to cope despite pain in a variety of situations (e.g., “I can enjoy things, despite the pain”). It is a 10-item instrument where items are scored on a range of 0 (not at all confident) to 6 (completely confident) for a total score range of 0–60 where higher levels indicate higher pain self-efficacy.

##### Mood

The Hospital Anxiety and Depression Scale (Zigmond and Snaith, 1983) was designed to screen for anxiety and depression in those with illness where symptoms may be conflated (e.g., aching muscles). The Hospital Anxiety and Depression Scale has a depression subscale and an anxiety subscale with 7 items each. Each item is scored on a scale of 0 to 3 relating to the frequency that a symptom has been experienced over the past 7 days, thus each subscale has a range of 0–21.

##### Fear of movement

The 13-item version of the Tampa Scale of Kinesiophobia (Miller et al., 1991) is a modified version of the original Tampa Scale of Kinesiophobia (TSK) where reverse-scored items were removed. The TSK was used to assess pain-related fear of movement. The TSK assesses pain-related fear beliefs (e.g., “Pain always means I have injured my body”) and fear of movement (e.g., “No one should have to exercise when he/she is in pain”) on a scale from 1 (strongly disagree) to 4 (strongly agree) resulting in a scale range from 13 to 52. Higher scores indicate higher fear of movement.

#### Daily Activity Levels

This study measured day-to-day minutes spent being active or sedentary. All the participants wore the accelerometers on their left wrist (this was the non-dominant hand for all but participant 1). A bout of physical activity was defined as 10 consecutive minutes of physical activity of any intensity. Given the study sample (i.e., people with chronic pain), the focus of this study was on measuring physical activity that occurred in daily life. Therefore, bouts of continuous physical activity of light (101–1,951 counts/minute), moderate (1,952–5,724 counts/minute) or vigorous (>5,725 counts/minute) intensity were included in the definition of physical activity, as calculated by the Freedson algorithm (Freedson et al., 1998). Sedentary bouts were defined as consecutive minutes (≥1 min) where there is <100 counts/min (Freedson et al., 1998). Physical activity in this study was operationalized as minutes spent in physical activity bouts and sedentary time was operationalized as minutes spent in sedentary bouts. Physical activity and sedentary time were treated as continuous variables.

#### Daily Diary Measures

##### Motivation to conserve resources

Motivation to conserve resources was measured with one item (“How important was it for you to conserve energy or strength today?”). This was measured on a scale from 1 (Not at all) to 5 (Very much).

##### Pain

Current pain intensity was rated on an 11-point Likert scale from 0 (no pain) to 10 (pain as bad as can be).

##### Self-regulatory fatigue

Self-regulatory fatigue was assessed by a three-item Self-regulatory Fatigue Scale short form (SRFS-3) developed in an unpublished PhD thesis (McMillan, 2019). The behavior, cognitive, and emotion facets of self-regulatory fatigue were measured by one item each from the behavior (“I have urges to hit, throw, break, or smash things”), cognitive (“I have no trouble making decisions”) and emotion subscales (“I get easily upset”). The items were measured on a 5-point Likert scale from 1 (Strongly disagree) to 5 (Strongly Agree). The item scores were summed to form a scale range from 3 to 15 where higher scores reflected higher self-regulatory fatigue.

##### Goal selection

Participants were presented with an item to assess which goal they would pursue each day (“Which goal is most important to you today?”). Participants could respond by selecting the goal they chose at the baseline meeting (see section “Baseline” below) or by selecting “other” and providing their daily goal response within a free-text box.

##### Goal self-efficacy

Goal self-efficacy was measured by up to four personalized self-efficacy items (Francis et al., 2004). The self-efficacy items were specific to the participant’s individual goal. One item assessed general confidence in the ability to achieve the goal (“I am confident I can pursue my goal today”) in all participants. Then, further items assessed confidence in ability to achieve the goal in the face of barriers of increasing difficulty. The barriers were also personal to each participant. Goal self-efficacy was measured with three or four items for each participant (depending on number of identified barriers) on a 5-point Likert scale from 1 (Not at all confident) to 5 (Completely confident), providing a score range of 1–20. The full list of additional goal self-efficacy items for each participant can be found in Supplementary Table 1.

##### Goal striving

Goal striving was measured with two items. One item measured goal efficiency (“How efficiently have you worked on your goal today?”) and was measured on a scale from 1 (Not at all) to 5 (Very much). One item measured goal pursuit frequency (“How often did you work on your goal today?”) on a scale from 1 (Not time at all) to 5 (All the time). The two items were summed to generate a score range from 1 to 10 where higher scores indicated higher goal striving.

##### Perceived demand

Perceived demand was measured with one item (“Overall, how demanding was your day?”) on a scale from 1 (Not at all) to 5 (Very much).

### Apparatus

Physical activity and sedentary time were measured using ActiGraph GT3X wearable accelerometer devices (ActiGraph GT3X; ActiGraph LLC, Pensacola, FL, United States). The GT3X collects raw tri-axial accelerometry data and takes measurements of wear time, energy expenditure, bouts of physical activity including duration and intensity of activity bout, metabolic rates, sedentary bouts, heart rate, an inclinometer which determines whether subjects are standing, sitting or lying down or if the device has been removed, and sleep activity. Accelerometers have demonstrated good reliability and validity in measuring physical activity (Eyler et al., 2003; Kelly et al., 2013).

A link to the daily diary was delivered via automated SMS text message to participants’ own smartphone (except in the case of participant one who did not have a smartphone and so was provided with one). Automated text messages were sent using a bulk SMS text message provider (Voodoo, 2020). Smartphones used in the study could be either Android or iOS operating systems. The smartphones were required to have 3G or 4G capability to ensure the diary could be completed without interruption. The text message provided a prompt to complete the diary.

### Procedure

#### Baseline

A brief semi-structured interview was conducted with each participant to illicit their valued activities, and to identify a goal and barriers, which were used to construct the personalized self-efficacy items. These interviews were conducted at the PMP (participant 1), at the University of Strathclyde (participant 2), in a public place chosen by the participant (participant 3) and at the participant’s home (participant 4). Participants then completed the baseline measures and were given a demonstration of how they would receive the daily diary and how to complete it. To reduce participant burden, measures of fear of movement and mood were not recorded by the researcher at the initial meeting as they were recorded at the first session of the pain management program by clinicians.

#### Pain Management Program

The PMP was delivered within a Scottish NHS secondary care setting by a multidisciplinary team (e.g., clinical psychologist, specialist nurse, and physiotherapist). The program was a weekly group intervention based on Acceptance and Commitment therapy (ACT) principles and included pain education, physiotherapy, pacing, acceptance, and mindfulness strategies as well as commitment to values and behavior change. Each participant engaged in the pain management program, which lasted either 10 or 12 weeks regardless of their participation in the research study.

#### Daily Diary Phase

The participants were provided the opportunity to complete the daily diary from the day following the baseline meeting, which was up to 1 week prior to the first day of the PMP. Completion of diary entries prior to the commencement of the PMP was to allow participants to get accustomed to the procedure, and so were not included in the analysis. The daily diary was completed online on the Qualtrics platform. A link to the diary was sent via a text message to participants’ smartphones at the agreed morning time. The morning diary included measures of pain intensity, goal identification, self-regulatory fatigue, goal striving, and goal self-efficacy. The evening diary, which was prompted by text message 12 h after the morning diary measured pain intensity, self-regulatory fatigue, perceived demand, and motivation to conserve resources. Additional morning and evening diary variables measured included mood, goal motivation, expected demand, expected progress and expected fatigue but these are not examined in this study. Every 2 weeks after beginning the diary phase, a face-to-face meeting was conducted at the site of delivery of the PMP to discuss any issues with the study, to ensure continued consent to participate, and to provide them with a new fully charged accelerometer. Participants were also encouraged to contact the researcher if any problems arose throughout the diary phase. After the diary phase was complete, a final face-to-face meeting was arranged to debrief the participant and provide the remuneration (£50 GBP) for their participation.

## Data Analysis

### Data Processing

Raw data were downloaded from the accelerometers and participants’ data files from each accelerometer were combined into one file for each participant. The downloaded raw data files were processed into epochs of 10 s using ActiLife software v6.13.3. Wear-time validation was conducted and a non-wear period was defined as 60 consecutive minutes of no activity using ActiLife software (Troiano et al., 2008). Bouts of physical activity and sedentary bouts were calculated by ActiLife software.

### Statistical Analysis

The data were analyzed individually for each participant using R statistical software v3.4.4. Missingness maps were produced for each participant using the AMELIA II package v1.7.5 (Honaker et al., 2011). Missingness maps were inspected visually to determine patterns of missingness. Where there was a very small number of daily diary observations missing at random (e.g., ≤0.05%), the mean of prior and subsequent observations was input. Otherwise, missing data was handled with multiple imputation using the AMELIA II package. The AMELIA II package uses an expectation-maximization bootstrapping (EMB) algorithm to model missing observations, specifically designed for time series data (Honaker et al., 2011). Five imputed datasets were produced where missing observations were imputed. As a bout of physical activity is defined as continuous movement for 10 min, imputed values <10 on physical activity were recoded to 0. All analysis was conducted on each of the five datasets and statistic estimates were calculated by pooling the results from each imputed dataset using Rubin’s rules (Rubin, 1996). Using Rubin’s rules to calculate parameter estimates accounts for the within and between variance of the combined results and calculating estimates with this method provides 95% confidence in inference when using multiply imputed datasets.

Time plots were examined for trends in the data. Autocorrelation, the correlation between a variable at the current time-point in a time series (*t*_0_) and the same variable at earlier time points or lags (e.g., where *t*-_1_ denotes one observation previous and *t*-_2_ denotes two observations previous), can arise when there are many repeated measurements of the same variables. Autocorrelograms were assessed for each variable to determine whether autocorrelation was present (Naughton and Johnston, 2014). Dynamic regression modeling was conducted to examine the relationship between the predictor variables and motivation to conserve resources, physical activity, and sedentary time. Using dynamic models to analyze N-of-1 data has been recommended because it is a flexible modeling approach (Vieira et al., 2017). Dynamic regressions can account for autocorrelation by including lags of the predictors and outcome variables as well as exogenous variables including trends in time and periodicity (e.g., morning and evening). Including lagged variables in the model which represent autocorrelation allows for independence between data points to be assumed. Dynamic regression models will not be formally described here as this has been done previously (Vieira et al., 2017).

Descriptive and multivariate analysis was conducted. As the purpose was to determine which variables had the most impact on motivation to conserve resources, physical activity and sedentary time, a stepwise approach was used to ascertain the model with the best model fit as determined by Akaike’s Information Criterion. Based on examination of the time plots and autocorrelograms, lags of the outcome variables, week, and weekday (i.e., whether it was a workday or weekend) were included as control variables as needed prior to the inclusion of predictor variables. The model residuals were then assessed for normality using a histogram and Q–Q plots and autocorrelation using autocorrelation function (ACF) plots and partial autocorrelation function (PACF) plots.

## Results

### Participant Characteristics

The participants’ demographic information, description of physical health condition(s) and baseline recordings of pain, self-regulatory fatigue, pain self-efficacy, fear of movement, mood and personal goal are shown in Table 1. Questionnaire scores for fear of movement, anxiety and depression for participant three are missing as this was not recorded at the first PMP session. Additional goals pursued by participants over the course of the study can be found in Supplementary Table 2. It should be noted that all participants chose a goal related to improving their emotional or social wellbeing.

**TABLE 1:** Baseline descriptive information for each participant.

	Participant 1	Participant 2	Participant 3	Participant 4
Age	48	41	50	59
Gender	Female	Male	Female	Female
Pain condition(s)	Neck, shoulder, and lower back pain	Arthritis, trapped nerve in neck, and diabetic neuropathy	Persistent pain	Osteoarthritis and polymyalgia rheumatica
Comorbid condition(s)	–	Diabetes type 1, retinopathy, nephropathy, high blood pressure, and angina	Suspected spastic paraplegia	Post viral depression
Pain duration	2–5 years	10–20 years	10–20 years	1–2 years
Current Pain intensity	5	9	6	5
Average pain intensity	7	8	10	8
Physical functioning	27	18	10	13
Self-regulatory fatigue	49	67	43	68
Pain self-efficacy	33	22	10	19
Fear of movement	27	19	–	36
Anxiety	6	16	–	8
Depression	11	12	–	9
Goal	Enjoy activities more	Manage emotions when unexpected setbacks arise	Improved management and maintenance of relationships	Feeling more confidence in managing pain
PMP length	10 weeks	12 weeks	12 weeks	10 weeks

*Scale ranges are as follows: current pain = 0–10; average pain intensity = 0–10; physical functioning = 8–40; self-regulatory fatigue = 18–90; pain self-efficacy = 0–60; fear of movement = 13–52; anxiety = 0–21; depression = 0–21.*

### Descriptive Statistics

**FIGURE 1:**
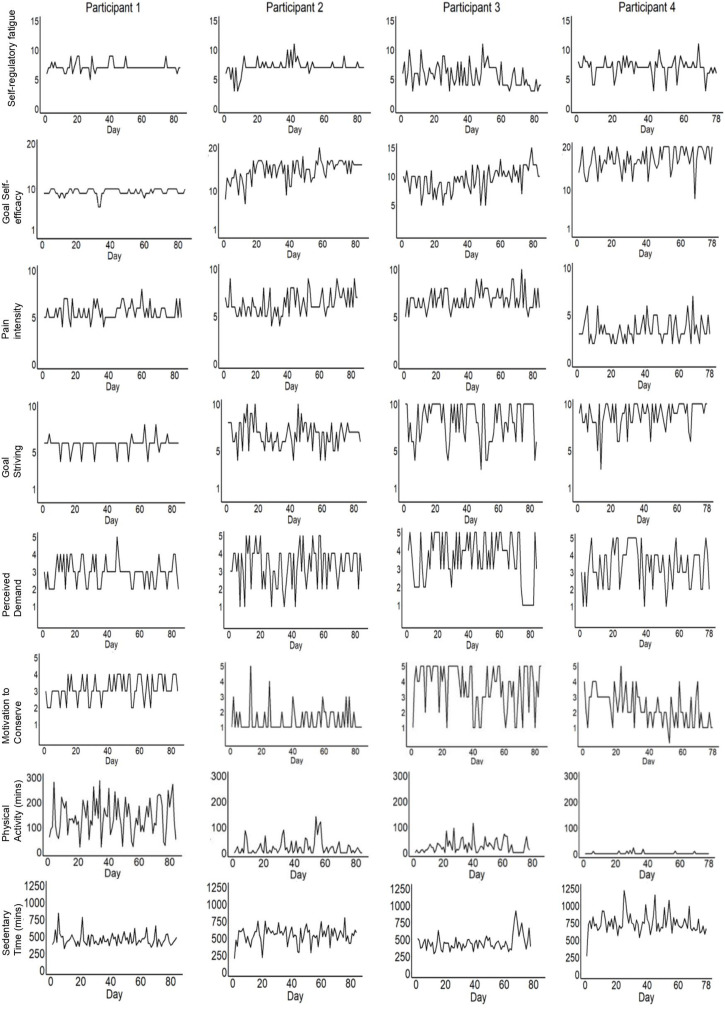
Time plots for all variables over time for each participant.

Compliance with diary completion was very high. Participants 2 and 3 completed 100% of diary entries and there were no missing observations. Participant 1 had one diary entry missing, meaning there was 0.006% of possible occasions and 0.05% of observations missing. Given the small amount of missing observations within the dataset for participant 1, the mean of the preceding and subsequent observations was inputted. Participant 3 had 1% observations missing as there was a technical issue with the accelerometer for the last 6 days of measurement. Participant 4 completed the diary on 97.5% of possible occasions and, overall, 4% of observations were missing. Evening observations were more likely to be missing than morning observations for participant 4. Therefore, multiple imputation was undertaken in participant 3 and 4’s data to provide full datasets. The results for participants 3 and 4, reported below, are the product of pooled estimates from five imputed datasets. Time plots of motivation to conserve resources, physical activity, sedentary time, and the predictor variables are shown in Figure 1.

Figure 1 illustrates that there is evidence of variance across participants and within participants over time on all variables. There may have been ceiling effects for participants 3 and 4 on goal striving and for participant 4 on self-efficacy. The means and standard deviations for physical activity, sedentary time, motivation to conserve resources, pain, self-regulatory fatigue, goal self-efficacy, goal striving and perceived demand for each participant are displayed in Table 2.

**TABLE 2:** Descriptive statistics of daily assessment of all study variables.

	Participant 1	Participant 2	Participant 3	Participant 4
Variable	*M* (SD)	*M* (SD)	*M* (SD)	*M* (SD)
Motivation to conserve	3.1 (0.7)	1.4 (0.8)	3.7 (1.4)	2.3 (1.0)
Physical activity (mins)	141.1 (66.4)	20.7 (29.1)	24.1 (24.4)	1.4 (4.4)
Sedentary time (mins)	453.2 (89.6)	541.3 (114.9)	455.6(113.0))	744.2 (143.4)
Pain	5.7 (0.9)	6.8 (1.2)	7.3 (1.1)	3.7 (1.3)
Self-regulatory fatigue	7.1 (0.7)	7.5 (1.2)	7.6 (1.7)	7.1 (1.5)
Goal self-efficacy	9.3 (0.8)	14.7 (2.4)	9.5 (2.1)	17.2 (2.7)
Goal striving	5.9 (0.7)	6.8 (1.3)	8.2 (2.0)	8.9 (1.4)
Perceived demand	3.0 (0.7)	3.3 (1.1)	3.6 (1.3)	3.5 (1.1)

*The possible goal self-efficacy score ranged from 1 to 15 for participant 3 and 1 to 20 for participants 1, 2, and 4.*

Dynamic regression models were conducted individually for each participant. Within each model, the reference measurement (*t*_0_) is either current morning or evening, depending on when the variable was measured. Pain intensity and self-regulatory fatigue were measured in both morning and evening diaries. The time of day of measurement is indicated in Table 3. Lag 1 (*t*-_1_) is the observation prior to *t*_0_, while lag 2 (*t*-_2_) is the observation prior to *t*-_1_. For example, lag 1 of variables measured in the evening (e.g., perceived demand) refers to the previous evening, while lag 1 of variables measured in the morning (e.g., goal self-efficacy) refers to the previous morning. We used autocorrelograms with ACF and PACF to guide the selection of the number of lags for predictors. It was unusual for there to be significant autocorrelation beyond lag 2. However, when significant autocorrelation of earlier lags (lag 3 onward) appeared to be present in autocorrelograms, these lags were included in models. When more recent lags were also accounted for within models (e.g., lag 0, lag 1, and lag 2), there was no effect of earlier lags (e.g., lag 3).

**TABLE 3:** Multivariate associations between predictor variables and outcomes in all participants.

	Participant			
Predictors	1	2	3	4
	**Motivation to conserve resources**			
Week	0.06***			
Weekday				
SRF (morn)	0.16* (lag 1)			
Goal striving (morn)	−0.25** (lag 0)		−0.24*** (lag 0)	−0.24*** (lag 0)
Goal self-efficacy (morn)				−0.08* (lag 0)
SRF (even)			0.31*** (lag 0)	
Pain (even)	0.18** (lag 0)		−0.39* (lag 0)	
Perceived demand (even)	0.22* (lag 0)		−0.24* (lag 0)	0.37*** (lag 0)
MCR (even)				0.24** (lag 1)
	**Physical activity**			
Week				
Weekday	88.61***			
Physical activity		0.43*** (lag 1)		
SRF (morn)				
Goal striving (morn)				
Goal self-efficacy (morn)				
SRF (even)				
Pain (even)				
Perceived demand (even)	24.65** (lag 2)			1.38*** (lag 0)
MCR (even)				
	**Sedentary Time**			
Week				
Weekday	−57.55**			
Sedentary time			0.51*** (lag 1)	
SRF (morn)	26.16* (lag 2)			
Goal striving (morn)		−22.49** (lag 0)		−34.27*** (lag 1)
Goal self-efficacy (morn)				
SRF (even)				
Pain (even)		−37.16*** (lag 0)		
Perceived demand (even)	−40.35** (lag 1)			−35.63*** (lag 1)
MCR (even)		31.05* (lag 0)	−18.09*** (lag 0)	

MCR, motivation to conserve resources; SRF, self-regulatory fatigue; morn, morning; even, evening. Lag 1 of morning variables refers to previous morning; lag 1 of evening variables refers to previous evening, etc. * p < 0.05, ** p < 0.01, and *** p < 0.001.

### Dynamic Regression Modeling Results

An overview of individual dynamic regression models of the effect of the pain, self-regulatory fatigue, goal self-efficacy, goal striving, and perceived demand on motivation to conserve resources, physical activity and sedentary time is displayed in Table 3.

In participant 1, the small positive association between week and motivation to conserve resources suggests that motivation to conserve resources increased slightly across the course of the study. In participant 1, motivation to conserve resources was higher on days when perceived demands, evening pain intensity, and previous morning self-regulatory fatigue were higher, and goal striving was lower. For participant 3, motivation to conserve resources was higher on days when goal striving, perceived demands, and evening pain intensity were lower, and evening self-regulatory fatigue was higher. In participant 4, motivation to conserve resources was higher on days when perceived demands and previous days’ motivation to conserve resources were higher, and goal striving, and goal self-efficacy were lower.

Physical activity was higher for participant 1 on the weekends and on days when perceived demands from 2 days’ previous were higher. Physical activity was higher for participant 2 on days when physical activity was higher the previous day. In participant 4, physical activity was higher on days when perceived demands were higher.

Sedentary time was higher for participant 1 on weekdays, when there was higher morning self-regulatory fatigue 2 days’ previously, and when the previous days’ perceived demands were lower. In participant 2, sedentary time was higher on days when motivation to conserve resources was higher, and goal striving and evening pain were lower. In participant 3, sedentary time was higher on days when sedentary time was higher the previous day and when motivation to conserve resources was higher. Sedentary time was higher for participant 4 on days when previous days’ perceived demand and pervious days’ goal striving were lower.

## Discussion

### Main Findings

The purpose of this study was to examine the interindividual motivational dynamics involved in motivation to conserve resources and activity levels over time in people with chronic pain. In line with the GRASSP model, the associations between the outcomes and goal-related and self-regulatory variables were unique across individuals. Goal striving and perceived demand were most frequently associated with outcomes across participants. Goal striving was related to less motivation to conserve resources (participants 1, 3, and 4) and less sedentary time (participants 2 and 4). Perceived demands were associated with higher motivation to conserve resources and physical activity, and lower sedentary time in two participants (1 and 4). Perceived demands were also associated with less motivation to conserve resources in another participant (participant 3). Higher self-regulatory fatigue predicted higher motivation to conserve resources (participants 1 and 3) and sedentary time (participant 1). Evening pain intensity was related to motivation to conserve resources, but in opposing directions (participants 1 and 3), and also to higher sedentary time (participant 2). The direction of the relationship between motivation to conserve resources and sedentary time was in opposing directions for two participants (2 and 3). Finally, goal self-efficacy was negatively associated with motivation to conserve resources in one participant (participant 4).

### Relationship to Past Research

The findings of this study are generally supportive of motivational accounts of activity patterns in people with chronic pain (Van Damme, 2014; Van Damme and Kindermans, 2015; Karoly, 2018) and previous research demonstrating that the context of a goal pursuit episode is associated with activity patterns (Karsdorp et al., 2010; Karsdorp and Vlaeyen, 2011; Schrooten et al., 2012; Van Damme et al., 2012; Pastor-Mira et al., 2019). The most consistent determinants of motivation to conserve resources and sedentary time in this study were goal striving and perceived demands. Perceived demands were also the only determinant of physical activity. The findings of this study are also partially in line with theory and past research asserting that people with chronic pain experience self-regulatory fatigue which negatively impacts self-regulatory performance by increasing motivation to conserve resources (Solberg Nes et al., 2010, 2011; Eisenlohr-Moul et al., 2013; Vervoort and Trost, 2016; Rost et al., 2017). In turn, motivation to conserve resources was related to sedentary time. Taken together, these findings suggest that there is a continuing evaluation of the costs and benefits of pursuing valued goals (Karoly, 2018; Van Damme et al., 2018).

However, no predictor variables were consistently associated with the outcomes across all participants. The direction of some observed relationships were contrary to expectations given previous research and theory (Karsdorp et al., 2010; Van Damme et al., 2012, 2018; Van Damme, 2014; Karoly, 2018). For example, while one participant reported higher motivation to conserve resources on days with higher pain and perceived demands (participant 1), the opposite associations were reported in another participant (participant 3). Further, there was a negative relationship between motivation to conserve resources and sedentary time in participant 3. The differences in the direction of relationships in this study are likely accounted for by whether physical activity levels were maintained despite pain, increased demands, and motivation to conserve resources (participant 1), or whether physical activity decreased due to motivation to conserve resources, meaning lower perceived demands and pain in the evening (participant 3). Meanwhile, goal self-efficacy was related to motivation to conserve resources in one participant, but it was generally not predictive of outcomes. This contrasts with past research demonstrating that self-efficacy predicts engagement in physical activity from groups-based studies (McAuley et al., 2011; Huffman et al., 2015).

Past evidence of the effect of self-regulatory fatigue and goal pursuit in people with chronic pain have often used experimental methods and retrospective self-report questionnaires and the average of group-aggregated data. The aggregation of group data can mask the direction of relationships within individuals (Johnston and Johnston, 2013; Yeo and Neal, 2013; McDonald et al., 2017a) and cannot account for the dynamic nature of self-regulatory processes. In addition, this study used objective measurement of physical activity and sedentary time with accelerometers as opposed to self-report measures. Self-report frequently results in biased estimation in people with chronic pain (Gosney et al., 2007; van Weering et al., 2011; Liu et al., 2014; Schaller et al., 2016), and for measurement at the individual level (Loney et al., 2011). The differences in the patterns of relationships observed in this study highlights the need to consider the individual goal-guided context (Karoly, 2018; Mun et al., 2019). The theory and methods used in this study have illustrated the heterogeneity in determinants of motivation to conserve resources and activity levels in people with chronic pain.

### Strengths and Limitations

Unlike most past research, the methods used in this study accounted for the dynamic nature of self-regulatory processes. Another strength of this study was the use of wearable accelerometer devices in conjunction with digital daily diaries. Previous diary studies in people with chronic pain have tended to assess either physical activity or the pursuit of personal goals but there is a lack of integration of both types of data (Van Damme, 2014). Additionally, using smartphones enabled participants to complete their dairy immediately after receiving the text message with the link to the diary. Studies which use paper-and pencil diaries can suffer from poor adherence and falsification of data and it is difficult to ascertain reliably the time at which they were completed (Stone et al., 2003; Broderick et al., 2008). Within this study, adherence was very high (the participant with the lowest adherence completed 96% of diary occasions). Furthermore, the use of N-of-1 observational methods and dynamic regression modeling allowed for models to be estimated for individuals over time while accounting for time trends and autocorrelation, thus reducing potentially biased estimates (Vieira et al., 2017).

Some limitations of the study should be noted. First, the pattern of relationships between the predictors and outcomes are unique to the individual participants and so different patterns may be observed in the future. Additionally, this study examined a limited number of goal related predictors and other self-regulatory, cognitive or affective processes may predict the outcomes in this population. The study measured some self-report variables retrospectively (e.g., motivation to conserve resources and perceived demand). As the nature of the study involved repeated measures within individuals, as opposed to measurement of a group, the reliability and validity of the self-report items used in this study is unknown. Preliminary data on the three item self-regulatory fatigue measure indicated that construct validity was acceptable but internal consistency was not satisfactory due to the low number of items while attempting to preserve the measurement of each subscale (McMillan, 2019). However, this data was from a student sample, not a sample of people with chronic pain, which may have affected interpretation of the items (Bonetti et al., 2001). We acknowledge that the unknown validity of single item, self-report measures and the three item self-regulatory fatigue measure is a limitation of this study. That said, it is the case that the longitudinal nature of data collection required a balance between the number of items and the need to reduce participant burden and the potential amount of missing data and participant retention in the study.

Additionally, while the purpose of the study was to examine factors which may affect activity levels during goal pursuit, goals chosen by participants were emotion regulation goals, not physical activity goals, and progress toward goal achievement was not measured in this study. Measuring goal progress may have provided further useful information about self-regulatory mechanisms. Future research, which uses ambulatory methods to measure the variables “in the moment” may be useful and provide more reliable estimate of relationships, as opposed to using retrospective items (Bentley et al., 2019).

### Implications for Methodology and Clinical Practice

Identifying the individual determinants of fluctuations in pain, motivation and self-regulatory processes will provide insight to people with chronic pain to enable them to manage to better their own condition and ultimately to pursue meaningful personal goals. Currently, psychological treatment programs evaluate whether the mean scores of psychosocial functioning indices have changed in the desired direction for groups of patients from pre to post intervention. For some patients, controlling fluctuations and reducing variability in pain and motivation may have a more significant impact on quality of life than changes from baseline scores (Mun et al., 2019; Winger et al., 2019). The effect of fluctuations or variability in pain and motivation are rarely assessed within treatment programs. This study has demonstrated that N-of-1 observational studies using accelerometers and digital daily diaries, where a link is delivered by text message, can be implemented with patients engaged in a pain management program. Further, it has been argued that N-of-1 trial designs could become the “gold standard” for assessing treatment efficacy (Bradbury et al., 2020) and could also be used to examine changes in variability in pain and motivation from pre to post intervention (Mun et al., 2019; Winger et al., 2019).

Evidence that fluctuations in self-regulatory fatigue, self-efficacy, pain, goal striving and perceived demands have differential effects on motivation to conserve resources, physical activity and sedentary time suggests that people with chronic pain would benefit from individualized treatment plans targeting motivational processes that affect the pursuit of their valued goals. For example, Acceptance and Commitment Therapy focuses on acceptance and mindfulness strategies as well as commitment to values and behavior change. Acceptance and mindfulness may increase self-regulatory capacity (Azam et al., 2016) while commitment to values may affect the cost-benefit analysis in undertaking activities. For the participants in this study, increasing self-regulatory capacity and goal striving, and decreasing the perceived demands of activities may result in more effective goal-directedness (Karoly, 2018).

It has been suggested that individually tailored activity pacing which takes into account the psychosocial context of activity, such as motivation for engagement in activity, is needed (Murphy, 2015; Mun et al., 2019). Data-driven tailored interventions to facilitate physical activity have been conducted previously with action planning and control cognitions in people with osteoarthritis (O’Brien et al., 2016). Further, a tailored, data driven activity pacing intervention which used accelerometer data reduced fatigue interference in those with osteoarthritis (Murphy et al., 2010).

The use of some digital health technologies and software can be expensive (e.g., Ecological Momentary Assessment platforms where cost for use of software, data storage on remote servers and cost per signal can be high), making it less accessible for some researchers. This study used a low-cost and easily implemented method of sending automated text messages using a bulk SMS provider, and the text messages included a link to the online digital diary. There are also free applications providing automated SMS schedulers which can be accessed from the Google Play Store and the Apple Store.

### Conclusion

This study demonstrated that the effect of self-regulatory fatigue, goal self-efficacy, goal striving and perceived demands on motivation to conserve resources, physical activity and sedentary time varied across participants. The observed relationships generally supported the GRASSP model which suggests that activity patterns in people with chronic pain can be accounted for by goal guided self-regulatory processes. This study illustrated that N-of-1 observational studies with digital health technologies can be conducted during pain management programs at low cost. The results from this study support the need for further research on within-individual variability of goal processes, the development of measures to support these research designs, and the development of individually tailored activity pacing interventions.

## Data Availability Statement

The datasets used within this study will be made available upon request to the corresponding author.

## Ethics Statement

The studies involving human participants were reviewed and approved by NHS South West-Central Bristol Research Ethics Committee. The patients/participants provided their written informed consent to participate in this study.

## Author Contributions

GM and DD contributed to the conception, design of the study, contributed to the manuscript revision, and read and approved the submitted version. GM recruited participants, collected data, conducted statistical analysis, and wrote the first draft of the manuscript. All authors contributed to the article and approved the submitted version.

## Conflict of Interest

The authors declare that the research was conducted in the absence of any commercial or financial relationships that could be construed as a potential conflict of interest. The reviewer RV declared a shared affiliation, with no collaboration, with one of the authors, DD, to the handling Editor at the time of review.
