# Supplementary material for: Self-Regulatory Processes, Motivation to Conserve Resources and Activity Levels in People With Chronic Pain: A Series of Digital N-of-1 Observational Studies
Source: Front Psychol. 2020 Sep 4;11:516485. doi: 10.3389/fpsyg.2020.516485 (PMC7499816; doi:10.3389/fpsyg.2020.516485)
Supplement: Supplementary file 1 [file Table_1.docx]

**Supplementary Table 1**

Full list of goal self-efficacy questions for each participant

| Participant | Item | Self-efficacy item |
| --- | --- | --- |
| 1 | **1.** | I am confident that I can pursue my goal today. |
|  | **2.** | I am confident that I can pursue my goal when I feel stressed |
|  | **3.** | I am confident that I can pursue my goal when I have low mood |
|  | **4** | I am confident that I can pursue my goal when I have long working hours |
|  |  |  |
| 2 | **1.** | I am confident that I can pursue my goal today. |
|  | **2.** | I am confident that I can pursue my goal when I have a flare-up of pain |
|  | **3.** | I am confident that I can pursue my goal when I feel low mood |
|  | **4.** | I am confident that I can pursue my goal when I feel anxious |
|  |  |  |
| 3 | **1.** | I am confident that I can pursue my goal today. |
|  | **2.** | I am confident that I can pursue my goal when I have a flare-up of pain |
|  | **3.** | I am confident that I can pursue my goal when I have work to do |
|  |  |  |
| 4 | **1.** | I am confident that I can pursue my goal today. |
|  | **2.** | I am confident that I can pursue my goal when I’m trying to push through pain |
|  | **3.** | I am confident that I can pursue my goal when I have a flare-up of extra pain |
|  |  | I am confident that I can pursue my goal when I feel low mood |

**Supplementary Table 2**

Full list of goals for each participant

| Participant | Goal | No. days pursued |
| --- | --- | --- |
| 1 | Enjoy activities more | 84 |
|  |  |  |
| 2 | Manage emotions when unexpected setbacks arise | 33 |
|  | To manage everything on my to-do list | 15 |
|  | Manage tasks of the day without feeling overwhelmed | 36 |
|  |  |  |
| 3 | Improved management and maintenance of relationships | 54 |
|  | Going for lunch | 8 |
|  | Shopping | 2 |
|  | Attend pain management programme | 5 |
|  | Shopping & lunch | 1 |
|  | Shopping & pain management | 2 |
|  | Go on holiday | 1 |
|  | Go to church | 3 |
|  | Go to church and dinner | 2 |
|  | Shopping and wedding reception | 1 |
|  | Attending a funeral | 2 |
|  | Hospital appointment | 1 |
|  | Hospital appointment & pain management | 1 |
|  | Hospital appointment & shopping | 1 |
|  | Visiting* | 1 |
|  | Podiatry and shopping | 1 |
|  | Appointments | 1 |
|  | Church and cinema | 1 |
|  |  |  |
| 4 | Feeling more confident in managing pain | 78 |

*This was the exact response entered by the participant
